# The Association of Increase of Human T-Cell Leukemia Virus Type-1 (HTLV-1) Proviral Load (PVL) With Infection in HTLV-1-Positive Patients With Rheumatoid Arthritis: A Longitudinal Analysis of Changes in HTLV-1 PVLs in a Single Center Cohort Study

**DOI:** 10.3389/fimmu.2022.887783

**Published:** 2022-05-06

**Authors:** Naoki Iwamoto, Takeshi Araki, Ayaka Umetsu, Ayuko Takatani, Toshiyuki Aramaki, Kunihiro Ichinose, Kaoru Terada, Naoyuki Hirakata, Yukitaka Ueki, Atsushi Kawakami, Katsumi Eguchi

**Affiliations:** ^1^Department of Immunology and Rheumatology, Division of Advanced Preventive Medical Sciences, Nagasaki University Graduate School of Biomedical Sciences, Nagasaki, Japan; ^2^Department of Rheumatology, Sasebo Chuo Hospital, Sasebo, Japan

**Keywords:** rheumatoid arthritis, human T-cell leukemia virus type 1, proviral load, infection, molecular-targeted therapy

## Abstract

**Objective:**

We evaluated changes of HTLV-1 proviral loads (PVLs) during treatment for rheumatoid arthritis (RA) and investigated whether these changes affect the clinical course in HTLV-1-positive RA patients.

**Methods:**

A total of 41 HTLV-1-positive RA patients were analyzed. Their clinical picture including disease activity [Disease Activity Score in 28 joints-erythrocyte sedimentation rate (DAS28-ESR), DAS28-CRP, simplified disease activity index (SDAI), and clinical disease activity index (CDAI)] and comorbidity were evaluated over a 2-year period. PVLs from peripheral blood mononuclear cells were investigated by real-time polymerase chain reaction (PCR). We investigated whether HTLV-1 PVLs is altered, or which clinical characteristics affect changes of HTLV1-PVLs during 2-year treatment.

**Results:**

Clinical disease activity was not changed during the 2-year observational period. The mean HTLV-1 PVL value change from baseline to 2 years was -1.2 copies/1000 PBMCs, which was not statistically significant. No baseline clinical characteristics influenced changes in HTLV-1 PVL. However, a numerical change of HTLV-1 PVLs was increased in 4 patients initiating the new biological/targeted synthetic disease-modifying antirheumatic drugs (b/tsDMARDs) at 2−10 months after starting the new b/ts DMARDs (numerical increase was 24.87 copies/1000 PBMCs). Infection occurred in 4 patients, and 3 of those patients showed an increased HTLV-1 PVL. Univariate analysis revealed an association between increase of HTLV-1 PVL and incidence of infection.

**Conclusions:**

Over 2 years, HTLV-1 PVL did not significantly change in our HTLV-1-positive RA patients. Individual changes in HTLV-1 PVL were correlated with incidence of infection but not disease activity which indicate that we may take precaution toward infection at the uptick of HTLV-1 PVL in HTLV-1-positive RA patients.

## Introduction

Rheumatoid arthritis (RA) is an autoimmune disease characterized by symmetrical joint inflammation that results in progressive joint destruction. Treatments efficacy for RA have been changed by biological and targeted synthetic disease-modifying antirheumatic drugs (b/tsDMARDs), currently, the use of which enables patients to achieve low diseases activity in up to 40% and remission in up to 20% (1). However, when using such immunosuppressive agents, the risk of infection must always be considered. Increasing numbers of infection-related adverse events with use of b/tsDMARDs have been reported, including reactivation of latent viruses (2).

Human T-cell leukemia virus type 1 (HTLV-1) is an exogenous retrovirus and the etiological agent of adult T-cell leukemia (ATL) and HTLV-1-associated myelopathy/tropical spastic paraparesis (HAM) (3). It infects 10-20 million people worldwide, with endemic regions in Japan, the Caribbean islands, south America and west Africa (4). In Japan, the nationwide estimate of the number of HTLV-1 carriers is at least 1.08 million, and the annual incidence of new infections by HTLV-1 is estimated at 3.8 per 100000 persons (5). HTLV-1 establishes lifelong latency in human T cells, and the lifetime risk of HTLV-1-infected individuals to develop ATL is estimated at 4 to 7% (6).

Since immunosuppressive agents can weaken host immunity, there have been questions as to whether treatment of HTLV-1 infected RA patients with immunosuppressive agents increases their risk of ATL/HTLV-1 associated disease development. Indeed, several cases in which ATL developed during treatment for RA have been reported. The patients in these cases had been treated by various DMARDs including methotrexate (MTX), abatacept and etanercept (7–10).

And, there is a possibility that HTLV-1 infection affects the clinical character of RA. Previously, we conducted a detailed investigation about the clinical features of HTLV-1-infected RA patients (11). That study revealed that the HTLV-1 proviral load (HTLV-1 PVL) was higher in patients with comorbidities of bronchiectasis, malignancies, and opportunistic infectious disease, as compared patients to without comorbidities. And there was no significant difference in HTLV-1 PVLs among types of b/tsDMARDs. However, that study was just a cross-sectional study. To elucidate the influence of HTLV-1 infection upon the incidence of other infections, which the cross-sectional study suggested to be related, and the influence of b/tsDMARDs on HTLV-1 PVL, a longitudinal study focused on changes of HTLV-1 PVL was needed. Here, we longitudinally measured HTLV-1 PVLs in daily clinical practice and analyzed the relations with the clinical course of RA including the incidence of infection and treatment response.

## Patients and Method

### Study Design and Patients

**Figure 1:**
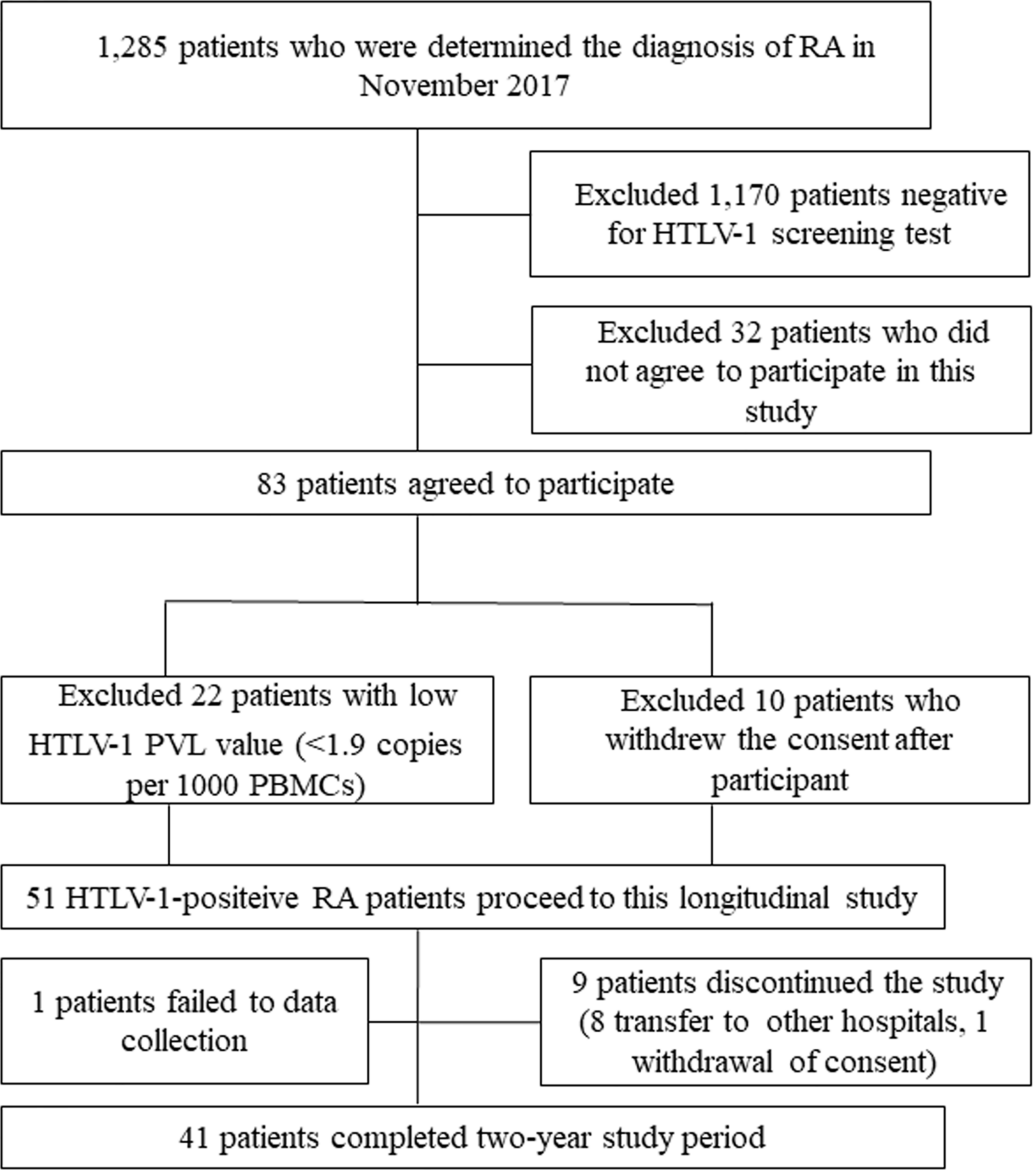
Flowchart for study sample. *RA*, rheumatoid arthritis; *HTLV-1 PVL*, Human T-cell leukemia virus type 1 proviral load; *PBMCs*, peripheral blood mononuclear cells.

This is a single center prospective study comprising HTLV-1-positive RA patients at Sasebo Chuo Hospital in the Nagasaki prefecture in Japan, conducted from December 2017 to April 2020. The patients gave their informed consent to be subjected to the protocol, which was approved by the Institutional Review Board of Sasebo Chuo Hospital (IRB approval no. 2017-23 and 2018-05). **Figure 1** show the study flowchart. The patients included in this analysis were enrolled in our previous study (11). In that study, we recruited 1285 patients whose diagnosis of RA had been made at Sasebo Chuo Hospital between December 2017 and April 2018. Among 1285 RA patients, 1170 patients negative on HTLV-1 screening as well as 32 HTLV-I-positive RA patients who did not agree to participate were excluded. As a result, the remaining 83 HTLV-I-positive RA patients participated the study. A chemiluminescent immunoassay (CLIA) (ARCHITECT^®^ HTLV, Abbott Japan, Tokyo, Japan) was used for an HTLV-1 screening test and a line immunoassay (LIA) (INNOLIA HTLV-1/11; Fujirebio, Europe NV, Belgium), was used for confirmation of HTLV-1 infection. Among these 83 patients, 22 patients were excluded because of low HTLV-1 PVL value [<1.9 copies per 1000 PBMCs; which indicated the lowest quartile level of PVLs in the previous study (11)] at baseline, and 10 patients did not agree to participate the study, remaining 51 patients who provided informed consent to participate in the present study were enrolled. Among enrolled patients 10 patients dropped out of the study because of 1 data missing, 8 transfer to other hospitals and 1 withdrawal of consent. Finally, we analyzed 41 patients in the study.

All patients had a diagnosis of RA based on the 1987 American College of Rheumatology (ACR) classification criteria for RA or the 2010 ACR/European League against Rheumatism (EULAR) classification criteria for RA. We collected the enrolled patients’ data at the time of the first measurement of HTLV1-PVL, including the disease-modifying antirheumatic drugs (DMARDs) use history, their disease duration, positivity of rheumatoid factor (RF) and anti-citrullinated protein antibodies (ACPA), and concomitant medications. The incidence of infection and development of HTLV-1-associated disease such as ATL were evaluated during a two-year follow-up period. Information about any changes of b/tsDMARDs was also collected during the study period.

### Clinical Assessment

The patients’ clinical disease activity was assessed using the Disease Activity Score in 28 joints-erythrocyte sedimentation rate (DAS28-ESR), the Disease Activity Score in 28 joints-C-reactive protein (DAS28-CRP), simplified disease activity index (SDAI), and clinical disease activity index (CDAI) at baseline and 6, 12, 18 and 24 months after the first measurement of HTLV-1 PVL. The incidence of infections reported by the patients as well as on the findings of physical examinations were also assessed.

### Quantification of HTLV-1 PVL

Genomic DNA was obtained from samples by standard proteinase K treatment. To quantify the proviral load, we performed real-time PCR as described previously (12). The primers for exon 3 of the HTLV-1 tax gene were 5’-GAAGACTGTTTGCCCACCACC-3’ and 5’- TGAGGGTTGAGTGGAACGGA-3’, and the probe was 5’-CACCGTCACGCTAACAGCCTGGCAA-3’. Genomic DNA (500 ng) was used for real-time PCR in a 50-μl reaction solution prepared with TaqMan Universal PCR master mix (Applied Biosystems, Foster City, CA). The amplification conditions were 50°C for 2 min, 95°C for 10 min, and then 40 cycles of 15 s at 95°C followed by 60 s at 60°C. All experiments were performed and analyzed using the ABIPRISM 7700 sequence detection system (Applied Biosystems). To measure cell equivalents in the input DNA, the recombination activating gene 1 (RAG-1) coding sequence in each sample was also quantified by real-time PCR. The sequences of the primers for RAG-1 exon 2 detection were 5’-CCCACCTTGGGACTCAGTTCT-3’ and 5’-CACCCGGAACAGCTTAAATTTC-3’, and the probe was 5’- CCCCAGATGAAATTCAGCACCACATA-3’. Amplification conditions were the same as those for the tax gene. The probes were labeled with fluorescent ‘6-carboxyfluorescein (reporter) at the 5’ end and fluorescent 6-carboxytetramethyl rhodamine (quencher) at the 3’ end. ATLL samples were analyzed in duplicate. The DNA of freshly purified ATLL cells, which harbor one copy of the HTLV-1 provirus, and the proviral load were used as positive controls. The proviral load was given the value of 100% when used as point of comparison.

### Statistical Analysis

GraphPad prism software (GraphPad Software, San Diego, CA) and JMP Statistical Software (SAS Institute, Cary, NC) were used for statistical analysis. Normal distribution of the data was confirmed using the Kolmogorov-Smirnov test. The distribution of baseline variables and proportion of disease activity in different patient subgroups were examined by Mann-Whitney U test and chi-square test. The Student’s paired t-test (for parametric data) or Wilcoxon signed rank test (non-parametric data) were used to detect statistically significant differences in HTLV-1 PVL change, inflammatory markers and disease activity. The Kruskal-Wallis test was used to compare baseline characteristics and disease course including incidence of infection and to estimate changes of disease activity among patient subgroups. Univariate analyses were used to identify factors contributing to the incidence of infection; our data were judged to be too few to perform multivariate analyses. All data are expressed as means ± standard deviations (SDs). P-values less than 0.05 were considered to indicate statistical significance.

## Results

### Baseline Characteristics

A total of 41 patients were analyzed in this study. The patients’ baseline demographic and clinical characteristics are summarized in **Table 1**. The mean age of patients was 67.9 ± 11.8 years, and most of the subjects were women (75.6%). The mean duration from RA onset to enrollment was 12.5 ± 10.4 years. The disease activity was relatively well controlled, i.e, the mean DAS28-ESR, DAS-CRP, SDAI and CDAI were 3.17 ± 1.09, 2.32 ± 0.90, 7.11 ± 6.23 and 6.96 ± 6.14, respectively. Except one patient, patients were prescribed MTX (n=34), b/tsDMARDs (n=10), and other conventional synthetic (cs) DMARDs (n=18). Concomitant use of an oral steroid was present in 17 patients.

**Table 1:** Clinical characteristics of the study population.

Female, n (%)	31 (75.6)
Age (years)	67.9 ± 11.8
Duration of RA (year)	12.5 ± 10.4
b/ts DMRDs use	9 (22.0)
Concomitant MTX use n, (%)	34 (82.9)
MTX dose (mg/week)	8.0 ± 2.15
Concomitant csDMARDs (except MTX) use n, (%)	18 (43.9)
Concomitant oral steroid use, n (%)	17 (41.5)
Oral steroid dose (mg/day)	3.74 ± 2.83
Biologic/ts DMARDs	
TNF inhibitors	4
IL-6 inhibitors	2
Abatacept	0
JAK inhibitors	4
ACPA positive, n (%)	26 (63.4)
RF positive, n (%)	28 (68.3)
DAS28-ESR	3.17 ± 1.09
DAS28-CRP	2.32 ± 0.90
SDAI	7.11 ± 6.23
CDAI	6.96 ± 6.14
HTLV-1 PVL (copies/1000 PBMCs)	44.8 ± 51.6

Data are means ± standard deviations.

RA, rheumatoid arthritis; b/tsDMARDs, biological and/or targeted synthetic disease-modifying antirheumatic drugs; MTX, methotrexate; csDMARDs, conventional synthetic disease-modifying antirheumatic drugs; TNF, tumor necrosis factor; IL-6, interleukin-6; JAK, Janus kinase; ACPA, anti-citrullinated protein antibodies; RF, rheumatoid factor; DAS, disease activity score; ESR, erythrocyte sedimentation rate; CRP, C-reactive protein; SDAI, simplified disease activity index; CDAI, clinical disease activity index activity; HTLV-1 PVL, Human T-cell leukemia virus type 1 proviral load; PBMCs, peripheral blood mononuclear cells.

### Clinical Course During Observational Period

Clinical disease activity as indicated by the DAS28-ESR, DAS28-CRP, SDAI, and CDAI, and the inflammatory markers were evaluated at baseline and 6, 12, 18 and 24 months after enrollment (**Table 2**). Although the disease activity showed a tendency to increase during the observational period, this increase was not statistically significant. Inflammatory markers did not change. Therapeutic regimens remained constant in almost all patients; however, new b/tsDMARDs (etanercept, tocilizumab and certolizumab pegol) were initiated in 3 patients (1 patient was DMARDs naïve and 2 patients had been treated by MTX) and the b/tsDMARD (tofacitinib) was switched in 1 patient (this patients had been treated by MTX). Infections were observed in 4 patients, and the infections were cystitis, pyelonephritis, sinusitis and cellulitis (onsets were 4-18months after enrollment, the causal agents were not identified, and one patient was hospitalized by cellulitis.). No ATL or other HTLV-1 related disease such as HAM developed in any patient during study period.

**Table 2:** The changes of disease activity and inflammatory markers.

	Baseline	6 months	12 months	18 months	24 months
ESR (mm/hr)	29.0 ± 25.8	27.8 ± 27.2	29.7 ± 27.1	27.1 ± 28.3	32.6 ± 27.7
CRP (mg/dl)	0.17 ± 0.18	0.14 ± 0.18	0.24 ± 0.46	0.29 ± 0.61	0.34 ± 0.65
DAS28-ESR	3.17 ± 1.09	2.90 ± 1.12	3.15 ± 1.03	3.24 ± 1.18	3.34 ± 1.26
DAS28-CRP	2.32 ± 0.90	2.15 ± 0.79	2.31 ± 0.76	2.52 ± 0.93	2.53 ± 1.09
SDAI	7.11 ± 6.23	6.22 ± 5.54	6.78 ± 5.10	8.54 ± 7.16	8.63 ± 7.76
CDAI	6.96 ± 6.14	6.10 ± 5.51	6.59 ± 5.09	8.29 ± 6.99	8.30 ± 7.44

No statistical significance was found in any disease activity and inflammatory markers at baseline vs those at 6,12, 18 and 24 months by the Wilcoxon signed rank test. Data are means ± standard deviations.

ESR, erythrocyte sedimentation rate; CRP, C-reactive protein; DAS, disease activity score; SDAI, simplified disease activity index; CDAI, clinical disease activity index activity.

### Changes in HTLV-1 PVL Value

**Figure 2:**
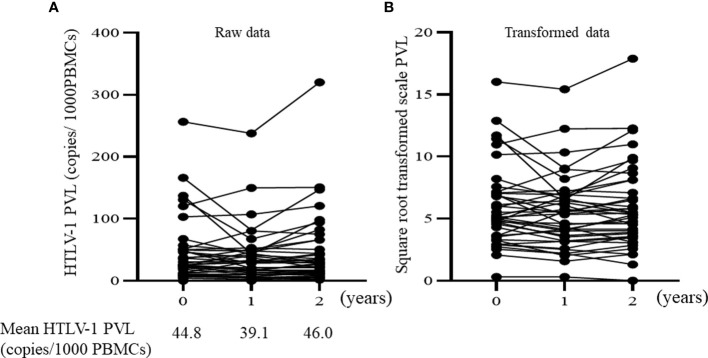
Time course of HTLV-1 PVL over 2 years. **(A)** Raw data of proviral load. The mean HTLV-1 PVL are expressed under the graph. **(B)** Square-root transformed values of the raw proviral load. *HTLV-1 PVL*, Human T-cell leukemia virus type 1 proviral load; *PBMCs*, peripheral blood mononuclear cells.

**Figure 2** illustrates the changes in HTLV-1 PVLs over the two-year study period (The square root transformed data for reducing the skewness of raw data are also illustrated). The mean HTLV-1 PVL values at baseline, 1 and 2 years after enrollment were 44.8, 39.1 and 46.0 copies per 1000 PBMCs, respectively. There were no statistically significant changes in HTLV-1 PVLs. We also analyzed the total lymphocyte count because the number of lymphocytes affects to the PVL values on PBMC. The lymphocyte subsets were no changed during study period (mean lymphocytes percentage of total white blood cells at baseline, 1 and 2 years after enrollment were 27.5, 28.3 and 28.6, respectively). In 24 patients, HTLV-1 PVLs increased over the 2 years whereas levels decreased in 16 patients. We explored the baseline characteristics that were associated with these two groups (PVLs increase or decrease) by performing univariate analysis (**Supplementary Table S1**). No baseline characteristics including b/ts DMARDs use was associated with changes of HTLV-1 PVLs. Regarding newly initiation of b/ts DMARDs, the mean HTLV-1 PVL values was increased after initiation of b/ts DMARDs. Four patients started new b/ts DMARDs during the study period without changing dose of concomitant oral steroid (newly introduction in 3 patients, switching in 1 patient, respectively as described above). The mean HTLV-1 PVL values of these 4 patients increased from 78.13 ± 108.2 copies/1000 PBMCs before initiating the new b/ts DMARD to 103.0 ± 149.0 copies/1000 PBMCs at about 2−10 months after starting the new b/ts DMARD.

### Comparison of Clinical Character and Clinical Course by Changes in HTLV-1 PVLs

To investigate whether changes in HTLV-1 PVLs affect the clinical course of RA during a two-year period, we divided the patients into quartile categories based on the change (Δ) in HTLV-1 PVL values from baseline at the 2-year point. Baseline characteristics investigated including b/tsDMARDs used at treatment and concomitant use of PSL were not different among these 4 groups (**Table 3**). No remarkable change of disease activity score during observation period was observed in any of the 4 groups, and changes were not different among the ΔHTLV-1 PVLs quartile groups. During the study period, infections were experienced by 4 patients. Three of the 4 patients were in Quantile 4 group (ΔHTLV-1 PVL from at 2 years to at baseline was >9.6 copies/1000 PBMCs). This result suggested that an increase of HTLV-1 PVL might indicate risk of infection.

**Table 3:** Comparison of baseline variables and clinical course by changes of HTLV-1 PVL values.

Frequency of subjects by changes of HTLV-1 PVL values (from at 2 years to baseline)	Quartile 1 (ΔHTLV-1 PVL:<-5.5 copies/1000 PBMCs)	Quartile 2 (ΔHTLV-1 PVL:-5.5-0.8 copies/1000 PBMCs)	Quartile 3 (ΔHTLV-1 PVL:0.8-9.6 copies/1000 PBMCs)	Quartile 4 (ΔHTLV-1 PVL:>9.6 copies/1000 PBMCs)	p-value (compared in all groups)
Number of patients	10	11	10	10	
Age (years)	72.2 ±14.4	65.9 ± 10.9	68.5 ± 12.1	65.5± 10.1	0.31
Duration of RA (year)	14.5 ± 10.9	12.0 ± 7.7	10.3 ± 8.1	13.5 ± 14.8	0.75
Concomitant oral steroid use n, (%)	5 (50.0)	5 (45.5)	3 (30.0)	4 (40.0)	0.82
Concomitant MTX use n, (%)	7 (70.0)	9 (81.8)	8 (80.0)	4 (40.0)	0.35
b/ts DMARDs use n, (%)	3 (30.0)	3 (27.3)	1 (10.0)	2 (20.0)	0.70
Changes of DAS28-ESR (end of study minus baseline)	0.08 ±1.26	0.22 ± 1.70	-0.10 ± 1.60	0.14 ± 1.01	0.95
Incidence of infection during 2 years n, (%)	0	0	1 (10.0)	3 (30.0)	<0.0001*

Data are means ± standard deviations.

HTLV-1 PVL, Human T-cell leukemia virus type 1 proviral load; RA, rheumatoid arthritis; MTX, methotrexate; b/tsDMARDs, biological and/or targeted synthetic disease-modifying antirheumatic drugs; DAS, disease activity score; ESR, erythrocyte sedimentation rate *P < 0.05.

### Factors Related to Infection in HTLV-1-Positive RA Patients

As revealed by above analysis, the changes in HTLV-1 PVL were correlated with the incidence of infection. To confirm this result, we next investigated which factors are specifically related with incidence of infection in HTLV-1-positive RA patients.

**Table 4** shows the factors associated with the incidence of infection during the study period in HTLV-1-positive RA patients in a univariate logistic analysis. Among the various factors such as b/tsDMARD used, PSL use and age, univariate analysis indicated that only the change of HTLV-1 PVL was associated with the incidence of infection.

**Table 4:** Univariate analysis for correlates of incidence of infection.

	OR	95% CI	p-value
Age (per 1-year increase)	1.003	0.918-1.097	0.94
Disease duration (per 1-year increase)	0.938	0.810-1.087	0.34
b/ts DMARDs use (yes/no)	0.788	0.074-8.432	0.84
Concomitant oral steroid use (yes/no)	0.438	0.042-4.609	0.49
Dose of oral steroid (Prednisolone) (per 1mg increase)	0.710	0.307-1.643	0.30
Changes in HTLV-1 PVL values: end of study minus baseline (per 1 increase)	1.059	1.002-1.120	0.026*

OR, odds raio; 95% CI, 95% confidence interval; b/tsDMARDs, biological and/or targeted synthetic disease-modifying antirheumatic drugs; HTLV-1 PVL, Human T-cell leukemia virus type 1 proviral load *P < 0.05.

## Discussion

We evaluated the clinical course of HTLV-1-positive RA patients and investigated the relation of that course with changes of HTLV-1 PVLs over a two-year period. The results of this study showed that disease activity was not associated with the status of HTLV-1 PVL, but the incidence of infection was associated with the increase of HTLV-1 PVL.

Overall, the mean value of HTLV-1 PVLs in this study was not significantly changed during the observational period. This result is similar with those of a previous report by Umekita et al. (13). They reported that the median HTLV-1 PVL value at baseline was 0.45 copies per 100 WBCs, and that value at two years after baseline was 0.46 copies per 100 WBCs (It has been estimated that values of PVL per WBCs is almost 2.5 fold lower as compared with that per PBMCs) (14). Although the mean value of HTLV-1 PVLs also did not significantly change, HTLV-1 PVLs did increase in some of our patients (Q4 in **Table 2**). Baseline characteristics including b/tsDMARD use and disease activity of those patients were not different from those of patients showing no increase of HTLV-1 PVL. However, after initiation of new b/tsDMARDs, HTLV-1 PVLs tended to increase in the present study. Considering these results, HTV1-PVL might increase in the short term (about 1-2 year) after the initiation of a b/tsDMARD and then level off. To confirm this hypothesis, we should investigate the time sequence analysis of HTLV-1 PVLs for a longer period in the future.

The infections were most frequently seen in the patients with increase of HTLV-1 PVL in this study. Moreover, univariable analysis indicated the increase of HTLV-1 PVL as a risk factor of infection in HTLV-1 PVL positive RA patients. HTLV-1 infection has been considered to contribute to the risk of infections because HTLV-1 causes T-cell dysfunction (15). For example, fewer numbers of naïve T lymphocytes in both CD4 + and CD8+ subpopulation were reported in HTLV-1 carriers (12). Experiments using an HTLV-1-infected mice model revealed that CD4+FOXp3+ regulatory T cells were increased and the IFN-γ response by CD8+ T Cells was limited due to PD-1 elevation (16). Furthermore, an increase in the CD4+CD25+ suppressor phenotype seen in HTLV-1-infected individuals was associated with an immunosuppressive state (17). Yanagihara et al. reported that expression of PD-1, PDL-1 on cytotoxic T lymphocytes was one possible mechanism for the high incidence of opportunistic infections in HTLV-1-infected patients (18). In fact, in clinical practice, high incidence rates of opportunistic infections such as strongyloidiasis, *P. jirovecii* pneumonia, tuberculosis and cytomegalovirus have been reported in HTLV-1-infected patients (19–23). High incidence of infection in HTLV-1-infected patients was also observed in RA. Hashiba et al. reported that the incidence rate of serious infection is higher in HTLV-1-positive RA patients compared to that in HTLV-1 negative RA patients (11.1 per 100 person-year *vs* 6.37 per 100 person-year, respectively) (24).

However, the relation of the change of PVL with the risk of infection was not elucidated by these previous studies. Our study is the first report to show an association between increase of HTLV-1 PVL and incidence of infection. This result suggests that T cell dysfunction caused by HTLV-1 infection becomes more significant as the HTLV-1 viral load increases. Our data showed that HTLV-1 PVLs increased after new b/tsDMARD initiation. Taken together, physicians should observe HTLV-1-positive RA patients with special caution for infection in the first 2 years or more after initiation of a b/tsDMARD.

No patient developed ATL or HTLV-1 related disease such as HAM, HTLV-1 uveitis in this study. Although immunosuppressive states have possibility to increase HTLV-1 PVL, it still remains unclear whether immunosuppressive agents affect the development of ATL in HTLV-1 carrier patients. In our previous study over a 24-week observational period, no ATL was developed among 50 HTLV-1-positive RA patients treated with a TNF-inhibitor and 27 HTLV-1-positive RA patients treated with a non-TNF-inhibitor (25, 26). Moreover, a 4-year observational study conducted by Umekita et al. also reported no ATL cases among HTLV-1-positive RA patients treated by various DMARDs (13). On the other hand, it has been reported that several patients developed ATL while receiving immunosuppressive treatments including bDMARDs (7, 9). In addition, development of ATL or HAM was observed after allogenic hematopoietic stem cell transplantation and renal transplantation who received immunosuppressive regime (27, 28). Several factors such as aging, high HTLV-1 PVL values, a family history, and a variety of genetic alterations that would relate to HTLV-1 oncogenesis have been reported as risk factors for development of ATL (5, 29). Besides immunosuppressive treatment, these risk factors might be more important for development of ATL in HTLV-1-positive RA patients. Thus, in the future we should investigate whether the immunosuppressive treatment shows a synergistic effect for development of HTLV-1-related disease in patients who have these risk factors.

Changes in HTLV-1 PVLs did not affect the disease activity of RA, and the disease activity of enrolled HTLV-1-positive RA patients was not significantly changed during the observational period. This result is similar with those of other studies. The above-mentioned 4-year observational study conducted by Umekita et al. showed no significant change of disease activity or HTLV-1 PVLs, and Endo et al. reported that the treatment response to non-TNF inhibitors was no different between HTLV-1 antibody-positive and -negative patients (13, 26). However, there is a possibility of lower efficacy of treatment, for instance with b/tsDMARDs, in HTLV-1-positive compared to -negative RA patients because HTLV-1 infection of T-cells and synovial fibroblasts has been shown to induce enhanced production of cytokines such as TNF-α, IL-1α (30–32). In fact, another cohort study reported reduced effectiveness of TNF inhibitors in HTLV-1 antibody-positive RA patients (25).

Several limitations of this study must be mentioned. The number of patients was small, and the number who initiated new b/tsDMARDs was even smaller. To confirm the tendency we found for the increment of HTLV-1 PVLs after initiation of b/tsDMARDs, a larger sample size is needed. Moreover, due to small number of infection cases, we could not perform multivariate logistic regression analysis to detect statistically related factors. Although this is the first study to show the possibility of increasing HTLV-1 PVL as a risk factor for infection, multivariable analysis with larger samples is important to rule out confounding factors. And we excluded the patients with low HTLV-1 PVL value because the PVL values in the patients with low PVL value at baseline had shown very little change during years in our previous experiences in clinical practice and we thought low PVL might less affect to clinical course of RA. So, the relation of changes in HTLV-1 PVL with clinical course of RA in RA patients with very low HTLV-1 PVL value remains unclear. Finally, with regard to the development of ATL, the study period of this study was relatively short. A very long latency period, as long as 50 years after HTLV-1 exposure, precedes ATL (33). Therefore, the duration of this study was insufficient for evaluating the risk of developing ATL.

In conclusion, our present study demonstrated that in most of our HTLV-1-positive RA patients, HTLV-1 PVLs did not significantly changed during treatment and the changes in HTLV-1 PVLs did not affect the treatment response. However, the increase of HTLV-1 PVL was suggested to be related to the incidence of infection; thus, we should take special precautions against infection when HTLV-1-positive RA patients show an uptick of their HTLV-1 PVL.

## Data Availability Statement

The raw data supporting the conclusions of this article will be made available by the authors, without undue reservation.

## Ethics Statement

The studies involving human participants were reviewed and approved by Sasebo Chuo Hospital. The patients/participants provided their written informed consent to participate in this study.

## Author Contributions

NI: conception and design of the study, analysis and interpretation of data, and drafting the article. KE: conception and design of the study, analysis and interpretation of data. NI, KE, and YU: collection and assembly of data. TA, AU, AT, ToA, KI, KT, YU, AK: analysis and interpretation of data, critical revision the manuscript. KE, YU, and AK: supervised the project. All authors contributed to the article and approved the submitted version.

## Conflict of Interest

The authors declare that the research was conducted in the absence of any commercial or financial relationships that could be construed as a potential conflict of interest.

## Publisher’s Note

All claims expressed in this article are solely those of the authors and do not necessarily represent those of their affiliated organizations, or those of the publisher, the editors and the reviewers. Any product that may be evaluated in this article, or claim that may be made by its manufacturer, is not guaranteed or endorsed by the publisher.

## Supplementary Material

Supplementary Figure 1 Time course of HTLV-1 PVL over 2 years in the patients who started new b/tsDMARDs. The Δ value of HTLV-1 PVL from at after starting new b/tsDMARDs to at before starting are expressed left side of the graph. + indicates the time point when new b/tsDMARDs was started. *HTLV-1 PVL.* Human T-cell leukemia virus type 1 proviral load; *b/tsDMARDs,* biological and/or targeted synthetic disease-modifying antirheumatic drugs; *PBMCs*, peripheral blood mononuclear cells.

## Abbreviations

RA, rheumatoid arthritis; b/tsDMARDs, biological and/or targeted synthetic disease-modifying antirheumatic drugs; HTLV-1, Human T-cell leukemia virus type 1; ATL, adult T-cell leukemia; HAM, HTLV-1-associated myelopathy/tropical spastic paraparesis; MTX, methotrexate; HTLV-1 PVL HTLV-1 proviral load; CLIA, chemiluminescent immunoassay; LIA, line immunoassay; ACR, American College of Rheumatology; EULAR, European League against Rheumatism; RF, rheumatoid factor; ACPA, anti-citrullinated protein antibodies; DAS28-ESR, Disease Activity Score in 28 joints-erythrocyte sedimentation rate; DAS28-CRP, Disease Activity Score in 28 joints-C-reactive protein; SDAI, simplified disease activity index; CDAI, clinical disease activity index; RAG-1, recombination activating gene 1; PBMCs, peripheral blood mononuclear cells; SDs, standard deviations; cs, conventional synthetic.

## References

[1] Aletaha D Smolen JS. Diagnosis and Management of Rheumatoid Arthritis: A Review. JAMA (2018) 320:1360–72. doi: 10.1001/jama.2018.13103 30285183

[2] Smolen JS Landewé R Bijlsma J Burmester G Chatzidionysiou K Dougados M. EULAR Recommendations for the Management of Rheumatoid Arthritis With Synthetic and Biological Disease-Modifying Antirheumatic Drugs: 2016 Update. Ann Rheum Dis (2017) 76:960–77. doi: 10.1136/annrheumdis-2016-210715 28264816

[3] Iwanaga M Watanabe T Yamaguchi K. Adult T-Cell Leukemia: A Review of Epidemiological Evidence. Front Microbiol (2012) 3:322. doi: 10.3389/fmicb.2012.00322 22973265 PMC3437524

[4] Proietti FA Carneiro-Proietti AB Catalan-Soares BC Murphy EL. Global Epidemiology of HTLV-I Infection and Associated Diseases. Oncogene (2005) 24:6058–68. doi: 10.1038/sj.onc.1208968 16155612

[5] Iwanaga M. Epidemiology of HTLV-1 Infection and ATL in Japan: An Update. Front Microbiol (2020) 11:1124. doi: 10.3389/fmicb.2020.01124 32547527 PMC7273189

[6] Satake M Yamada Y Atogami S Yamaguchi K. The Incidence of Adult T-Cell Leukemia/Lymphoma Among Human T-Lymphotropic Virus Type 1 Carriers in Japan. Leuk Lymphoma (2015) 56:1806–12. doi: 10.3109/10428194.2014.964700 25219595

[7] Nakamura H Ueki Y Saito S Horai Y Suzuki T Naoe T. Development of Adult T-Cell Leukemia in a Patient With Rheumatoid Arthritis Treated With Tocilizumab. Intern Med (2013) 52:1983–6. doi: 10.2169/internalmedicine.52.0468 23994996

[8] Shiraishi T Ishimoto H Akata K Kawanami T Yatera K Mukae H. An Autopsy Case Report of Adult T-Cell Leukemia Accompanied by Rheumatoid Arthritis Mimicking Diffuse Panbronchiolitis. J UOEH (2017) 39:55–61. doi: 10.7888/juoeh.39.55 28331122

[9] Takajo I Umekita K Ikei Y Oshima K Okayama A. Adult T-Cell Leukemia/Lymphoma as a Methotrexate-Associated Lymphoproliferative Disorder in a Patient With Rheumatoid Arthritis. Intern Med (2018) 57:2071–5. doi: 10.2169/internalmedicine.0308-17 PMC6096007 29491299

[10] Okamoto M Eguchi K Hida A Terada K Aramaki T Nonaka F. Development of Adult T-Cell Leukaemia/Lymphoma During the Treatment of Rheumatoid Arthritis. Modern Rheumatol Case Rep (2019) 3:87–91. doi: 10.1080/24725625.2018.1549932

[11] Eguchi K Iwanaga M Terada K Aramaki T Tuji Y Kurushima S. Clinical Features and Human T-Cell Leukemia Virus Type-1 (HTLV-1) Proviral Load in HTLV-1-Positive Patients With Rheumatoid Arthritis: Baseline Data in a Single Center Cohort Study. Mod Rheumatol (2020) 30:471–80. doi: 10.1080/14397595.2019.1602931 30938551

[12] Yasunaga J Sakai T Nosaka K Etoh K Tamiya S Koga S. Impaired Production of Naive T Lymphocytes in Human T-Cell Leukemia Virus Type I-Infected Individuals: Its Implications in the Immunodeficient State. Blood (2001) 97:3177–83. doi: 10.1182/blood.v97.10.3177 11342446

[13] Umekita K Hashiba Y Kariya Y Kubo K Miyauchi S Aizawa A. The Time-Sequential Changes of Risk Factors for Adult T-Cell Leukemia Development in Human T-Cell Leukemia Virus-Positive Patients With Rheumatoid Arthritis: A Retrospective Cohort Study. Mod Rheumatol (2019) 29:795–801. doi: 10.1080/14397595.2018.1519890 30246572

[14] Matsumoto C Sagara Y Sobata R Inoue Y Morita M Uchida S. Analysis of HTLV-1 Proviral Load (PVL) and Antibody Detected With Various Kinds of Tests in Japanese Blood Donors to Understand the Relationship Between PVL and Antibody Level and to Gain Insights Toward Better Antibody Testing. J Med Virol (2017) 89:1469–76. doi: 10.1002/jmv.24802 28252206

[15] Mitsuya H Guo HG Cossman J Megson M Reitz MS Jr Broder S. Functional Properties of Antigen-Specific T Cells Infected by Human T-Cell Leukemia-Lymphoma Virus (HTLV-I). Science (1984) 225:1484–6. doi: 10.1126/science.6206569 6206569

[16] Espindola OM Siteur-van Rijnstra E Frankin E Weijer K van derVelden YU Berkhout B. Early Effects of HTLV-1 Infection on the Activation, Exhaustion, and Differentiation of T-Cells in Humanized NSG Mice. Cells (2021) 10:2514–31. doi: 10.3390/cells10102514 PMC8534134 34685494

[17] Montes M Sanchez C Verdonck K Lake JE Gonzalez E Lopez G. Regulatory T Cell Expansion in HTLV-1 and Strongyloidiasis Co-Infection Is Associated With Reduced IL-5 Responses to Strongyloides Stercoralis Antigen. PloS Negl Trop Dis (2009) 3:e456. doi: 10.1371/journal.pntd.0000456 19513105 PMC2686100

[18] Yanagihara T Ikematsu Y Kato K Yonekawa A Ideishi S Tochigi T. Expression of PD-1 and PD-L1 on Cytotoxic T Lymphocytes and Immune Deficiency in a Patient With Adult T Cell Leukemia/Lymphoma. Ann Hematol (2018) 97:359–60. doi: 10.1007/s00277-017-3146-z 28967040

[19] Verdonck K González E Van Dooren S Vandamme AM Vanham G. Human T-Lymphotropic Virus 1: Recent Knowledge About an Ancient Infection. Lancet Infect Dis (2007) 7:266–81. doi: 10.1016/S1473-3099(07)70081-6 17376384

[20] Tanaka T Sekioka T Usui M Imashuku S. Opportunistic Infections in Patients With HTLV-1 Infection. Case Rep Hematol (2015) 2015:943867. doi: 10.1155/2015/943867 26693362 PMC4674586

[21] Schierhout G McGregor S Gessain A Einsiedel L Martinello M Kaldor J. Association Between HTLV-1 Infection and Adverse Health Outcomes: A Systematic Review and Meta-Analysis of Epidemiological Studies. Lancet Infect Dis (2020) 20:133–43. doi: 10.1016/S1473-3099(19)30402-5 31648940

[22] Kawano N Nagahiro Y Yoshida S Tahara Y Himeji D Kuriyama T. Clinical Features and Treatment Outcomes of Opportunistic Infections Among Human T-Lymphotrophic Virus Type 1 (HTLV-1) Carriers and Patients With Adult T-Cell Leukemia-Lymphoma (ATL) at a Single Institution From 2006 to 2016. J Clin Exp Hematop (2019) 59:156–67. doi: 10.3960/jslrt.18032 PMC6954174 31866618

[23] Ashida C Kinoshita K Nozaki Y Funauchi M. Fatal Outcome in a Patient Under Immunosuppressant Therapy Infected With Human T-Lymphotropic Virus Type 1 (HTLV-1), Cytomegalovirus (CMV) and Strongyloides Stercoralis: A Case Report. BMC Infect Dis (2020) 20:470. doi: 10.1186/s12879-020-05195-0 32615937 PMC7331000

[24] Hashiba Y Umekita K Kimura M Iwao C Iwao K Kariya Y. High Incidence of Serious Infections Requiring Hospitalisation in Human T-Cell Leukaemia Virus Type 1-Positive Rheumatoid Arthritis: A Case-Controlled Observational Study. Modern Rheumatol (2021) 1–9. doi: 10.1093/mr/roab077 34897491

[25] Suzuki T Fukui S Umekita K Miyamoto J Umeda M Nishino A. Brief Report: Attenuated Effectiveness of Tumor Necrosis Factor Inhibitors for Anti-Human T Lymphotropic Virus Type I Antibody-Positive Rheumatoid Arthritis. Arthritis Rheumatol (2018) 70:1014–21. doi: 10.1002/art.40461 29471588

[26] Endo Y Fukui S Umekita K Suzuki T Miyamoto J Morimoto S. Effectiveness and Safety of Non-Tumor Necrosis Factor Inhibitor Therapy for Anti-Human T-Cell Leukemia Virus Type 1 Antibody-Positive Rheumatoid Arthritis. Mod Rheumatol (2021) 31:972–8. doi: 10.1080/14397595.2020.1847802 33161771

[27] Yamauchi J Yamano Y Yuzawa K. Risk of Human T-Cell Leukemia Virus Type 1 Infection in Kidney Transplantation. N Engl J Med (2019) 380:296–8. doi: 10.1056/NEJMc1809779 30650320

[28] Kawano N Yoshida S Kawano S Kuriyama T Tahara Y Toyofuku A. The Clinical Impact of Human T-Lymphotrophic Virus Type 1 (HTLV-1) Infection on the Development of Adult T-Cell Leukemia-Lymphoma (ATL) or HTLV-1-Associated Myelopathy (HAM) / Atypical HAM After Allogeneic Hematopoietic Stem Cell Transplantation (Allo-HSCT) and Renal Transplantation. J Clin Exp Hematop (2018) 58:107–21. doi: 10.3960/jslrt.18011 PMC6408177 30089749

[29] Kogure Y Kataoka K. Genetic Alterations in Adult T-Cell Leukemia/Lymphoma. Cancer Sci (2017) 108:1719–25. doi: 10.1111/cas.13303 PMC5581529 28627735

[30] Fukui S Nakamura H Takahashi Y Iwamoto N Hasegawa H Yanagihara K. Tumor Necrosis Factor Alpha Inhibitors Have No Effect on a Human T-Lymphotropic Virus Type-I (HTLV-I)-Infected Cell Line From Patients With HTLV-I-Associated Myelopathy. BMC Immunol (2017) 18:7. doi: 10.1186/s12865-017-0191-2 28158970 PMC5292003

[31] Sakai M Eguchi K Terada K Nakashima M Yamashita I Ida H. Infection of Human Synovial Cells by Human T Cell Lymphotropic Virus Type I. Proliferation and Granulocyte/Macrophage Colony-Stimulating Factor Production by Synovial Cells. J Clin Invest (1993) 92:1957–66. doi: 10.1172/JCI116789 PMC288362 8408648

[32] Araya N Sato T Ando H Tomaru U Yoshida M Coler-Reilly A. HTLV-1 Induces a Th1-Like State in CD4+CCR4+ T Cells. J Clin Invest (2014) 124:3431–42. doi: 10.1172/JCI75250 PMC4109535 24960164

[33] Bangham CRM. Human T Cell Leukemia Virus Type 1: Persistence and Pathogenesis. Annu Rev Immunol (2018) 36:43–71. doi: 10.1146/annurev-immunol-042617-053222 29144838

