# Supplementary figures and images for: The Association of Increase of Human T-Cell Leukemia Virus Type-1 (HTLV-1) Proviral Load (PVL) With Infection in HTLV-1-Positive Patients With Rheumatoid Arthritis: A Longitudinal Analysis of Changes in HTLV-1 PVLs in a Single Center Cohort Study
Source: Front Immunol. 2022 May 6;13:887783. doi: 10.3389/fimmu.2022.887783 (PMC9120818; doi:10.3389/fimmu.2022.887783)

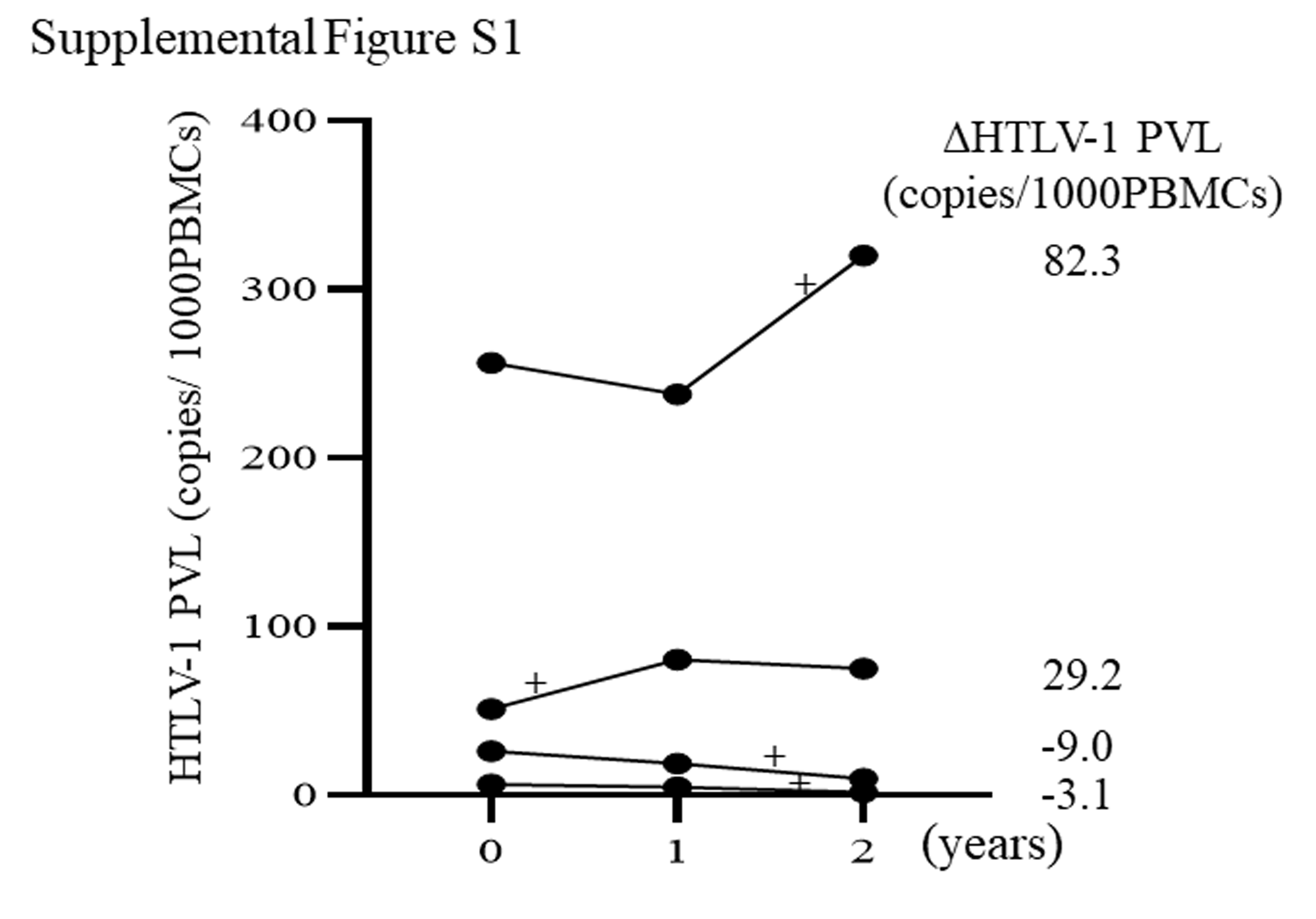

Supplement: Supplementary Figure 1 — Time course of HTLV-1 PVL over 2 years in the patients who started new b/tsDMARDs. The Δ value of HTLV-1 PVL from at after starting new b/tsDMARDs to at before starting are expressed left side of the graph. + indicates the time point when new b/tsDMARDs was started. HTLV-1 PVL. Human T-cell leukemia virus type 1 proviral load; b/tsDMARDs, biological and/or targeted synthetic disease-modifying antirheumatic drugs; PBMCs, peripheral blood mononuclear cells. [file Image_1.tif]
