# Supplementary material for: The Association of Increase of Human T-Cell Leukemia Virus Type-1 (HTLV-1) Proviral Load (PVL) With Infection in HTLV-1-Positive Patients With Rheumatoid Arthritis: A Longitudinal Analysis of Changes in HTLV-1 PVLs in a Single Center Cohort Study
Source: Front Immunol. 2022 May 6;13:887783. doi: 10.3389/fimmu.2022.887783 (PMC9120818; doi:10.3389/fimmu.2022.887783)
Supplement: Supplementary file 2 [file Table_1.docx]

**Supplemental Table S1.**

**Univariate analysis for correlates of increase of HTLV-1 PVL during 2-year study period**

|  | OR | 95% CI | p-value |
| --- | --- | --- | --- |
| Age  (per 1-year increase) | 0.967 | 0.913-1.023 | 0.23 |
| Disease duration  (per 1-year increase) | 0.982 | 0.924-1.04387 | 0.55 |
| b/ts DMARDs use  (yes/no) | 0.376 | 0.095-1.494 | 0.16 |
| MTX use  (yes/no) | 4.583 | 0.770-27.296 | 0.08 |
| Concomitant oral steroid use (yes/no) | 0.675 | 0.191-2.382 | 0.54 |
| Dose of oral steroid (Prednisolone)  (per 1mg increase) | 0.867 | 0.673-1.117 | 0.26 |

*OR* odds raio, *95% CI* 95% confidence interval, b*/tsDMARDs* biological and/or targeted synthetic disease-modifying antirheumatic drugs, *MTX* methotrexate
